# Fever management in children and insights into fever of unknown origin: a survey among Italian pediatricians

**DOI:** 10.3389/fped.2024.1452226

**Published:** 2024-11-01

**Authors:** Elena Chiappini, Michela Orlandi, Alberto Chiarugi, Antonio Di Mauro, Antonella Insalaco, Gregorio Paolo Milani, Monica Vallini, Andrea Lo Vecchio

**Affiliations:** ^1^ Department of Health Sciences, Pediatric Infectious Diseases Unit Meyer Children’s University Hospital, IRCCS, University of Florence, Florence, Italy; ^2^ Department of Health Sciences, University of Florence, Florence, Italy; ^3^ Department of Health Sciences, Section of Clinical Pharmacology and Oncology Headache Center, University of Florence, Florence, Italy; ^4^ Pediatric Primary Care, National Health Care System, ASL BA, Bari, Italy; ^5^ Division of Rheumatology, IRCCS Ospedale Pediatrico Bambino Gesù, ERN-RITA Center, Rome, Italy; ^6^ Pediatric Unit, Fondazione IRCCS Ca’ Granda Ospedale Maggiore Policlinico, Milan, Italy; ^7^ Department of Clinical Sciences and Community Health, University of Milan, Milan, Italy; ^8^ Pediatric Primary Care, National Health Care System, AUSL Bologna Città, Bologna, Italy; ^9^ Department of Translational Medical Sciences, University of Naples Federico II, Naples, Italy

**Keywords:** fever, fever of unknown origin, children, paracetamol, ibuprofen

## Abstract

**Background:**

Fever is a common symptom in children, but despite existing guidelines, pediatricians may not fully apply recommendations. Fever of Unknown Origin (FUO) is generally referred to as an unexplained prolonged fever. However, a standardized FUO definition and management is missing.

**Objective:**

To collect updated data on the approach to fever and FUO among Italian pediatricians.

**Methods:**

A cross-sectional anonymous survey was conducted among a large sample of primary care and hospital pediatricians. The panel group formulated and proposed a practical FUO definition, using a modified Delphi approach. A 75% consensus was required to reach an agreement.

**Results:**

Among 620 respondents, paracetamol was the first-choice antipyretic for 97.7% of participants, followed by ibuprofen; 38.4% prescribed antipyretics based on a specific body temperature rather than on child's discomfort, while physical methods were almost completely abandoned. Alternate treatment was recommended by 19.8% (123/620) of participants, 16.9% (105/620) would prescribe antipyretics to prevent adverse events following immunization. Regarding FUO diagnosis, 58.3% (362/620) considered as cut-off a body temperature above 38°C; the duration required was one week according to 36.45% (226/620) of participants, two weeks according to 35.32% (219/620). The FUO definition proposed by the expert panel reached 81% of consent. Large agreement was observed on first-level laboratory and instrumental investigations in the diagnostic evaluation of FUO, whereas more discrepancies arose on second and third-level investigations. Compared to what participants reported for the treatment of non-prolonged fever, a significant decrease in the prescription of paracetamol as first-choice drug in children with FUO was observed (80.5%; *P* < 0.0001). Interestingly, 39% of participants would empirically recommend antibiotics, 13.7% steroids, and 4.5% Nonsteroidal Anti-Inflammatory Drugs (NSAIDs) for persistent FUO.

**Conclusion:**

Non-recommended behaviors in fever management persist among pediatricians, including alternating use of paracetamol and ibuprofen, and their prophylactic use for vaccinations. Our data confirm the variability in the definition, work-up, and management of FUO. We observed that in children with FUO paracetamol was significantly less commonly preferred than in non-prolonged fever, which is not supported by evidence. Our findings combined with evidence from existing literature underlined the need for future consensus documents.

## Introduction

Fever is a common symptom in children, most commonly due to self-limiting viral infections, and it is a benign defensive mechanism against pathogens (1). However, its persistence in pediatric patients can cause concerns among caregivers.

Fever of Unknown Origin (FUO) is a challenge for pediatricians and may be an alarming situation for caregivers. Furthermore, it constitutes a considerable burden for the healthcare system, accounting for 3% of pediatric hospitalizations in the United States (2).

The classic definition of FUO was coined in 1961 by Petersdorf and Beeson in a prospective study including a series of adult patients presenting with documented fever (temperature above 38.0°C) of at least 3 weeks duration, without a diagnosis despite a week of hospital investigations (3). However, the definition of FUO in the pediatric population is not standardized: over the years, numerous studies have focused on defining FUO in pediatrics, proposing various body temperature thresholds and fever duration periods. Most studies have required a minimum duration of 2 to 3 weeks to classify a fever as FUO (4–8). With the advancement of diagnostic techniques, most of the common causes of FUO from the past can now be more rapidly ruled out, thus leading to a shortened number of fever days before considering FUO diagnosis. More recently, some authors defined FUO as a body temperature higher than 38.0°C lasting at least 8 days or more, with negative history, physical examination, and preliminary investigations (1).

The etiology of FUO in children has a wide spectrum, though it can be summarized into four main categories: infectious, neoplastic, inflammatory, and miscellaneous causes (including metabolic disorders, drug fever and factitious fever). A large systematic review of case series of FUO in children reported that 51% of cases were ultimately due to infections, 9% to collagen vascular disease and 6% to malignancy; notably, 23% of FUOs remain undiagnosed despite thorough investigations (9). However, proportions largely vary among different studies (10–12). The etiologies of FUO notably differ between high-income countries and low- and middle-income countries (LMICs). While infections remain the most common cause across all regions, the specific types of infections vary (1). In high-income countries, over the last decade, with the advent of novel diagnostic assays [i.e., polymerase chain reactions (PCR)], and improved understanding of the pathogenesis of atypical viral and bacterial infection and of autoimmune/autoinflammatory conditions, the rate of reported undiagnosed FUO cases has decreased (12). Additionally, the management of FUO in pediatric patients, encompassing both diagnostic evaluations and pharmacological treatments, lacks standardization, which may lead to the excessive use of laboratory and radiological examinations.

To the best of our knowledge, no existing study has determined whether paracetamol or ibuprofen is preferable for children with FUO during the diagnostic work-up. On this issue, it should be taken into account that while paracetamol primarily acts as an antipyretic, ibuprofen also exerts an anti-inflammatory effect, which may potentially interfere with underlying conditions and the diagnostic process (13).

“Fever phobia” is a term first coined in the 1980 by Barton Schmitt (14) to describe misconceptions of parents about fever, although subsequent studies over the years have demonstrated its presence also among healthcare professionals (15, 16). This “phobia” may be amplified in case of prolonged and unexplained fever, both in caregivers and pediatricians, therefore leading to inappropriate treatment of fever before a diagnosis is reached.

A study based on a questionnaire administered to 562 Italian pediatricians was conducted in 2018 (17) to investigate changes in knowledge and misconceptions about fever, six years after the release of the Italian guidelines for the management of fever (IFG) (18): the study results indicated a progressive improvement in the management of fever. However, several incorrect practices persisted, including the use of physical methods (6.4%), administering antipyretics without considering the presence of discomfort (61.8%), and alternating between different antipyretics (12.3%) (17).

We conducted a further survey on a similar cohort of Italian pediatricians to gather updated information about their approach to children with fever, specifically focusing on the use of antipyretic medications and their knowledge and handling of FUO.

Various studies have proposed different diagnostic workups for the investigation of children with FUO (10, 12, 19), though no standardized algorithm is currently available. One of the aims of our survey was to underline the variability in the management of FUO among pediatricians, and to obtain data on diagnostic approaches in the clinical practice, that might be incorporated in future studies for the elaboration of guidelines of this complex issue.

## Materials and methods

Under the patronage of Italian Society of Pediatrics, a cross-sectional survey was conducted among 18.000 pediatricians, mostly members of the Society, between 20th November 2023 and 20th January 2024.

Pediatricians received an invitation to take part in the survey through an e-mail containing an explanation of the study scope and design, and a link to answer an anonymous questionnaire**.**

In the invitation, pediatricians were explained that questions referred to the management of fever in children, with particular focus on FUO. Each questionnaire was filled out anonymously after reading the information on privacy regulations in accordance with the European Union Regulation number 79/2016 (20) and obtaining consent to the processing of data necessary for the study.

### Questionnaire development, administration, and data collection

The survey was developed by a panel of 7 Italian pediatricians working in academic institutions, hospitals, or community settings with more than 10 years of clinical experience in the management of fever in children, active participation in boards and committees of the Italian Society of Pediatrics or other scientific societies, and international publications on this topic. The panel comprised two pediatric infectious disease specialists, one pediatric rheumatologist, one pediatric emergency medicine expert, one pharmacologist and three general pediatricians (the authors of the current study). A pilot test was run among all the panel members and modified according to their observations.

The final questionnaire consisted in 37 questions, 31 closed-ended questions (with only a single answer allowed), and 6 open-ended questions, including 3 questions on demographic information of participants. In order to avoid missing data, it was mandatory to answer any question to proceed further in the questionnaire. Questions were divided into three main sections to collect the following information:

(1) age group, geographic origin, work setting and years of work experience of survey participants;
(2) use of antipyretics in the clinical practice, specifically about choice of antipyretic drug (paracetamol or ibuprofen), indications and possible contraindications to their use.
(3) knowledge of FUO among pediatricians, in terms of definition, diagnostic work-up and treatment, with additional questions about the number of FUO cases observed in their clinical practice; an additional scope of the last part of the questionnaire was to find a definition of FUO largely shared by participants.

The full questionnaire is reported in Supplementary Appendix A. Ethics approval was not required for this type of study.

### Data analysis and statistics

Answers to the survey were automatically collected into an electronic database and then transferred into an Excel spreadsheet. Results were expressed as absolute frequency and percentage, when needed 95% confidence intervals (95% CIs) were calculated. Categorical data were compared using *χ*2 tests, or the Fisher exact test, as appropriate. SPSS software package (SPSS; IBM Corporation; version 26) was used and a *P*-value < 0.05 was considered as statistically significant.

Using a modified Delphi approach, the multidisciplinary panel of 7 experts reviewed the literature and formulated one possible practical definition for FUO. Participants were invited to score the definition by the following scale: 1, strong disagreement; 2, fair disagreement; 3, no opinion; 4, fair agreement; 5, full agreement. For the analysis of results, responses were categorized as negative (score 1–2) or neutral (score 3), and positive (score 4–5). The percentage of voters who gave positive responses were considered, and the cut-off level for consensus was 75% (21).

This project was supported by an unrestricted grant from Angelini SpA, who played no role in this manuscript.

## Results

### Participants characteristics

A total of 620 out of the 18.000 members submitted the questionnaire (response rate 3.4%). Table 1 shows the demographic characteristics of the study participants. As regards their work setting, 50% (311/620) were hospital pediatricians, 41.3% (256/620) were primary care pediatricians, and 8.5% (53/620) worked in other settings (i.e., private practices, private clinics).

**Table 1:** Characteristics of the survey participants.

Age group	*N* (%)
26–39	197 (31.0)
40–49	101 (16.3)
50–59	115 (18.6)
60–69	185 (29.9)
70–83	22 (3.6)
Geographic provenance	
Northern Italy	328 (52.9)
Center Italy	144 (23.3)
Southern Italy	148 (23.9)
Work setting	
Primary care pediatrician	256 (41.3)
Hospital pediatrician	311 (50.0)
Other	53 (8.5)
Years of work experience as pediatrician	
0–10	239 (38.9)
11–20	88 (14.0)
21–30	133 (21.5)
>30	160 (25.9)

### Choice of antipyretic drug

Paracetamol was considered the first-choice antipyretic drug by 97.7% (606/620) of participants, with only 2.0% (12/620; *P* < 0.0001) preferring ibuprofen, while two out of 620 pediatricians (0.3%) declared to use the two drugs interchangeably. In children not respondent to the first-choice drug, 38.6% (234/606) of those who preferred paracetamol would continue prescribing it verifying and adjusting the dosage as needed, 38.3% (232/606) would switch to ibuprofen as a second-choice drug, and 19.1% (116/606) would recommend alternating paracetamol and ibuprofen. Among the smaller group who initially prescribed ibuprofen, in cases of persistent fever, 58.3% (7/12) would recommend alternating the two antipyretics, while 33.3% (4/12) would continue with ibuprofen, checking and adjusting the dosage if necessary. Only one out of 12 would switch to paracetamol.

### Indications for the use of antipyretics

The use of antipyretics based on the presence of discomfort, rather than a specific body temperature cut-off, was recommended by 61.6% (382/620) of pediatricians. The remaining participants (38.4%; 238/620) recommended using antipyretics only above a specific body temperature cut-off.

Specifically, 69.5% (178/256) of primary care pediatricians vs. 56.0% (204/364) of hospital pediatricians/other prescribed antipyretics based on the presence of discomfort (*P* = 0.0007). Regarding the use of antipyretics in children undergoing vaccinations, 82.4% (512/620) of participants would not recommend administering paracetamol or ibuprofen prophylactically. Interestingly, 16.9% (105/620) would prescribe paracetamol preemptively post-vaccination to reduce the incidence of fever or local reactions. Of these, 42% (44/105) would recommend it after any vaccination, while 58% (61/105) would recommend it exclusively after specific vaccines: most commonly, 16% (17/105) after the Meningococcal B vaccine (MenB) and 13.3% (14/105) after the Measles, Mumps, Rubella, and Varicella vaccine (MMR/MMRV).

In case of fever and discomfort following immunization, 95.3% (591/620) of respondents would prescribe paracetamol, 0.81% (5/620) would prescribe ibuprofen, and 3.55% (22/620) would not recommend any drug.

### Contraindications and bacterial superinfections

According to 85.3% (529/620) of participants, certain clinical conditions contraindicate the use of ibuprofen: the most frequent were suspected/confirmed impaired renal function (398/620, 62.7%), moderate or severe dehydration (362/620, 58%) and age below 3 months (353/620, 56.9%). Additionally, 46.9% (291/620) would not recommend paracetamol in specific clinical situations, with the most frequent contraindication (254/620, 40.9%) being chronic hepatopathy.

### Definition of FUO

The questionnaire reported four definitions of FUO from current literature (9) and participants were asked to choose the most appropriate one according to their opinion and clinical experience. For most participants (49%; 304/620), FUO was defined by the persistence of fever for more than 8 days in a child whose medical history, examination, and laboratory tests do not reveal a possible cause (Table 2).

**Table 2:** Definition of FUO according to survey participants.

	Participants (%, *n*)
A. Fever (> 38°C) persisting for at least 3 weeks, in a patient who underwent routine medical investigations exclusively in a hospital setting, without the cause being determined	15 (95/620)
B. Two or more weeks of fever (>38.5°C), measured on at least 4 different occasions	4.7 (29/620)
C. Febrile illness for which the cause could not be clarified during at least 3 weeks of outpatient evaluation, or for more than one week of hospital observation	28.9 (179/620)
D. Persistence of fever for more than 8 days in a child whose medical history, examination, and laboratory tests do not reveal a possible cause	49 (304/620)
Definition proposal formulated by the panel	Agreement (4–5 on a 1-to-5 Likert scale)
Continuous fever, with daily peaks above 38°C, without apparent explanation, documented by a healthcare professional * for at least two consecutive weeks. *Fever should be documented by a healthcare professional on multiple occasions in hospital setting, or at least three times in different days in outpatient setting.	81.1 (504/620)

Secondly, the following definition of FUO elaborated by the board panel was presented: “Continuous fever, with daily peaks above 38°C, without apparent explanation, documented by a healthcare professional, for at least two consecutive weeks. Fever should be documented by a healthcare professional on multiple circumstances in a hospital setting or at least three times in different days in outpatient setting” (Table 2). Respondents who gave a rating between 1 and 3 on the Likert scale could successively answer an open-ended question to express their doubts about the proposed definition: the most frequent observation (17/83, 20.4%) concerned the duration of fever, with two weeks being considered too long to define FUO. Nevertheless, large agreement was reached, since 81.1% (504/620) of participants gave a 4 or 5 answer on the Likert scale, with no difference between primary care pediatricians (209/256;81.6%) and hospital pediatrician/other (294/364; 80.8%; *P* = 0.785).

When asked about the number of cases of FUO they encountered during the previous year in their clinical practice, 61% (378/620) of participants declared they diagnosed either 1 or no case of FUO, 29.5% (183/620) 2 to 5 cases, 6.9% (43/620) 6 to 10 cases and 2.6% (16/620) 10 or more cases.

Primary care pediatricians significantly more often declared to diagnose zero or one case per year (191/256; 74.6% *vs*. 153/364; 42.0%; *P* < 0.0001).

### Diagnostic approach to FUO

**Figure 1:**
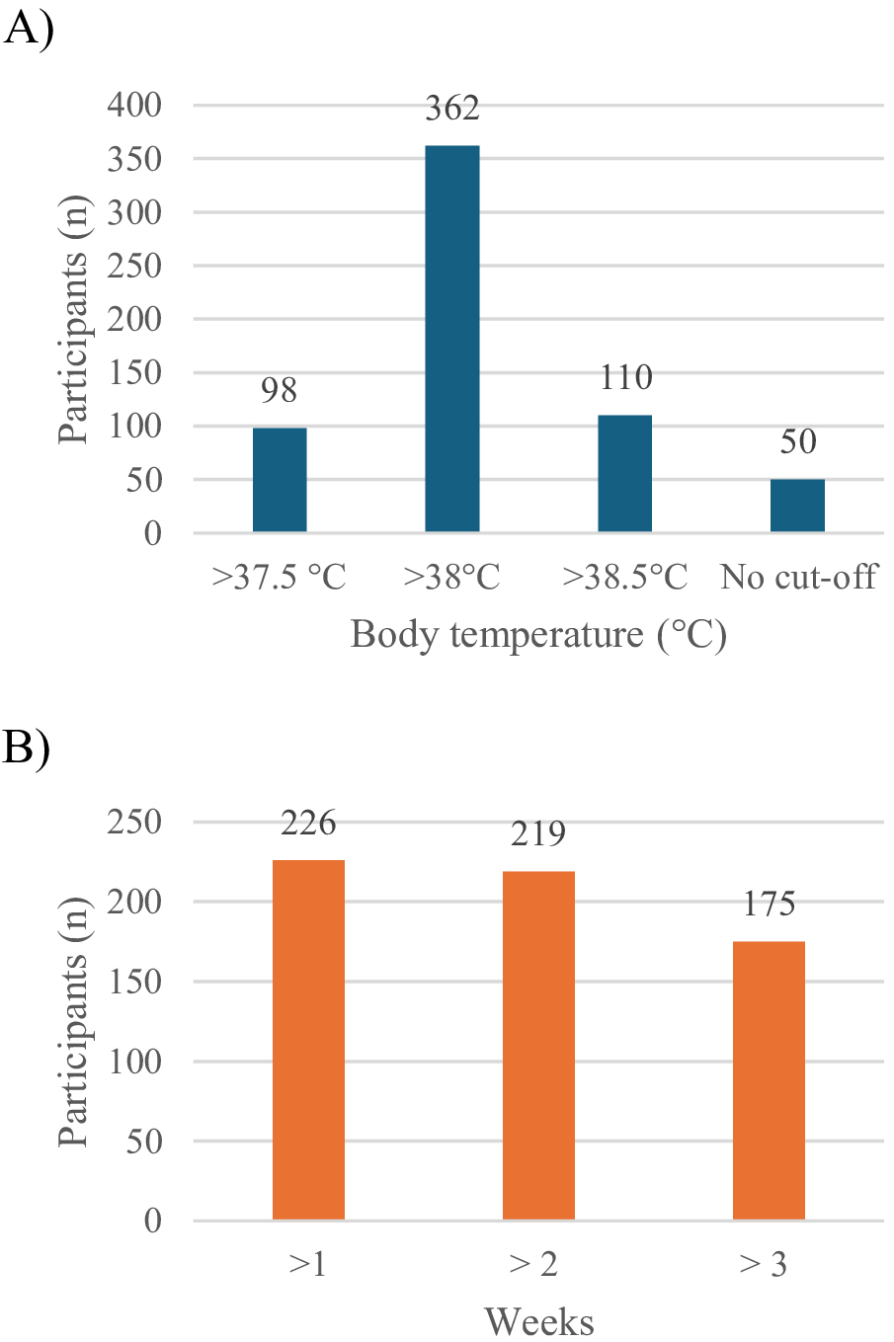
Criteria for the diagsnosis of FUO according to survey participants. **(A)** Body temperature cut-off. **(B)** Fever time duration (weeks).

According to the participants, the main diagnostic criteria for FUO were body temperature measured by a healthcare provider on multiple occasions, and the duration of fever: 58.3% (362/620) considered as cut-off for defining the presence of fever a body temperature above 38°C (Figure 1A); the duration of fever required for defining FUO was one week according to 36.45% (226/620) of participants, two weeks according 35.32% (219/620) (Figure 1B).

The appropriate setting for body temperature measurement and clinical evaluation of the child, according to 66.1% (410/620) of the participants, might be both an outpatient and hospital setting; 19% (118/620) stated that the evaluation should be undertaken exclusively in a hospital setting; 13.4% (83/620) exclusively in an outpatient setting. However, 60% (372/620) would eventually recommend hospitalization to document more accurately the presence and characteristics of prolonged fever.

Participants were questioned about the investigations they would prescribe in a child with FUO, after collecting a detailed medical history and conducting a complete objective examination. More specifically, they indicated what laboratory, instrumental tests and specialist evaluations they would consider appropriate in the diagnostic work-up, specifying also whether they would request them as first-level or second/third-level assessments.

**Figure 2:**
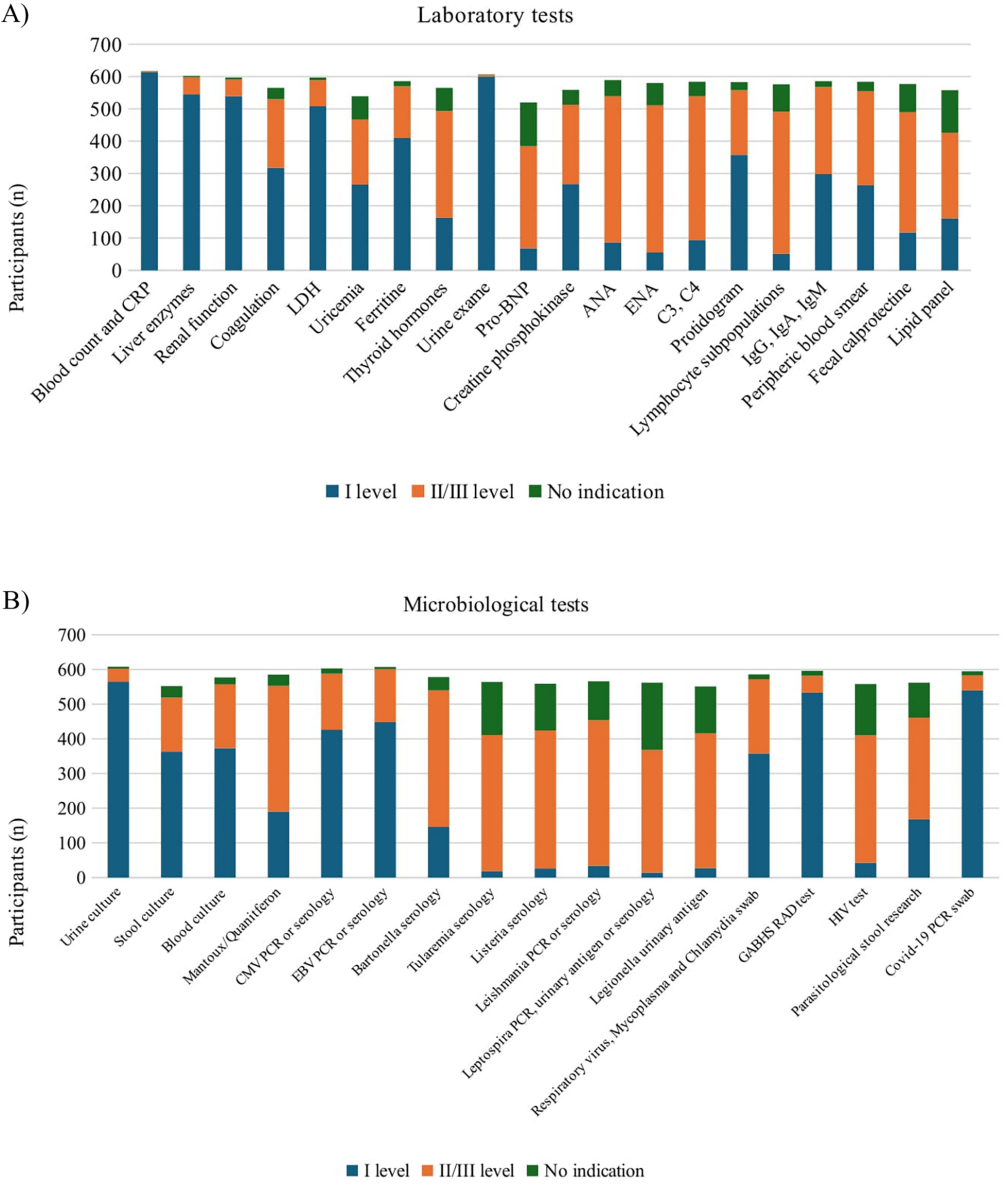
Investigations recommended by survey participants. **(A)** Laboratory tests. CBC, complete blood count; CRP, C-reactive protein; LDH, lactate dehydrogenase; ANA, anti-nuclear antibodies; ENA, extractable Nuclear Antigen Antibodies. **(B)** Microbiological tests. CMV, cytomegalovirus; EBV, epstein-barr virus; GABHS, group A beta-hemolytic Streptococcus; RAD, rapid antigen detection; HIV, human immunodeficiency virus.

As first-level laboratory tests, 99% (614/620) would prescribe complete blood count (CBC) and C-reactive protein (CRP), 96.7% (600/620) urine exam, 87% (545/620) liver enzymes, 86.9% (539/620) renal function, 81.9% (508/620) lactate dehydrogenase (LDH), 66% (410/620) ferritine. As second-level exams, 73% (453/620) would test anti-nuclear antibodies (ANA), 73.5% (456/620) extractable nuclear antigen antibodies (ENA), 71.7% (445/620) complement components (C3, C4) and 70.9% (440/620) lymphocyte subsets (Figure 2A).

Regarding microbiological investigations, as first-level tests 91.1% (565/620) would prescribe urine culture, 60.1% (373/620) blood culture and 58.5% (363/620) stool culture. Furthermore, 85.9% (533/620) would prescribe a rapid antigen detection test (RAD) for group A beta-hemolytic *Streptococcus pyogenes* (GABHS), 87% (540/620) Covid-19 PCR on nasopharyngeal swab, 72.2% (442/620) Epstein-Barr Virus (EBV) serology or PCR, 68.7% (426/620) Cytomegalovirus serology or PCR and 58% (358/620) respiratory virus*, Mycoplasma pneumoniae* and *Chlamydia pneumoniae* PCR on nasopharyngeal swab. As second-level infectious disease tests, 67.7% (420/620) of participants would prescribe Leishmania serology or PCR, 64.1% (398/620) Listeria serology, 63.5% (394/620) Tularemia and Bartonella serology (Figure 2B).

**Figure 3:**
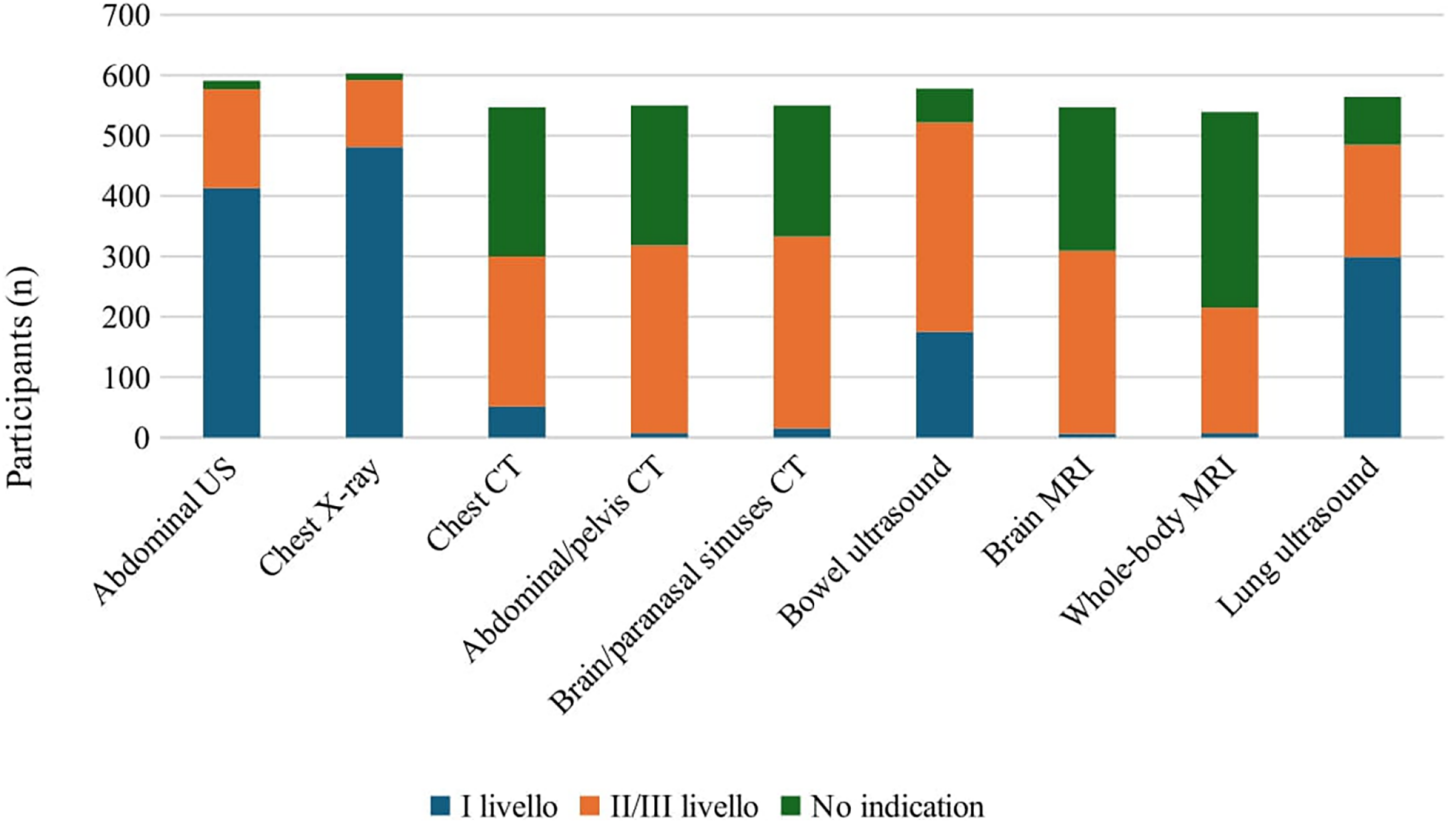
Instrumental tests recommended by survey participants. US, ultrasound; CT, computed tomography; MRI, magnetic resonance imaging.

As first-level instrumental investigations, 77.5% (481/620) would request chest x-Ray and 66.6% abdominal ultrasound. Notably, 48.2% (299/620) would request a lung ultrasound. As second-level investigations 55.9% (347/620) would prescribe bowel loops ultrasound to rule out Inflammatory Bowel Disease (IBD, 53.3% (331/620) chest CT, 51.2% (318/620) brain and paranasal sinuses CT and 50.1% (311/620) abdominal and pelvic CT (Figure 3).

**Figure 4:**
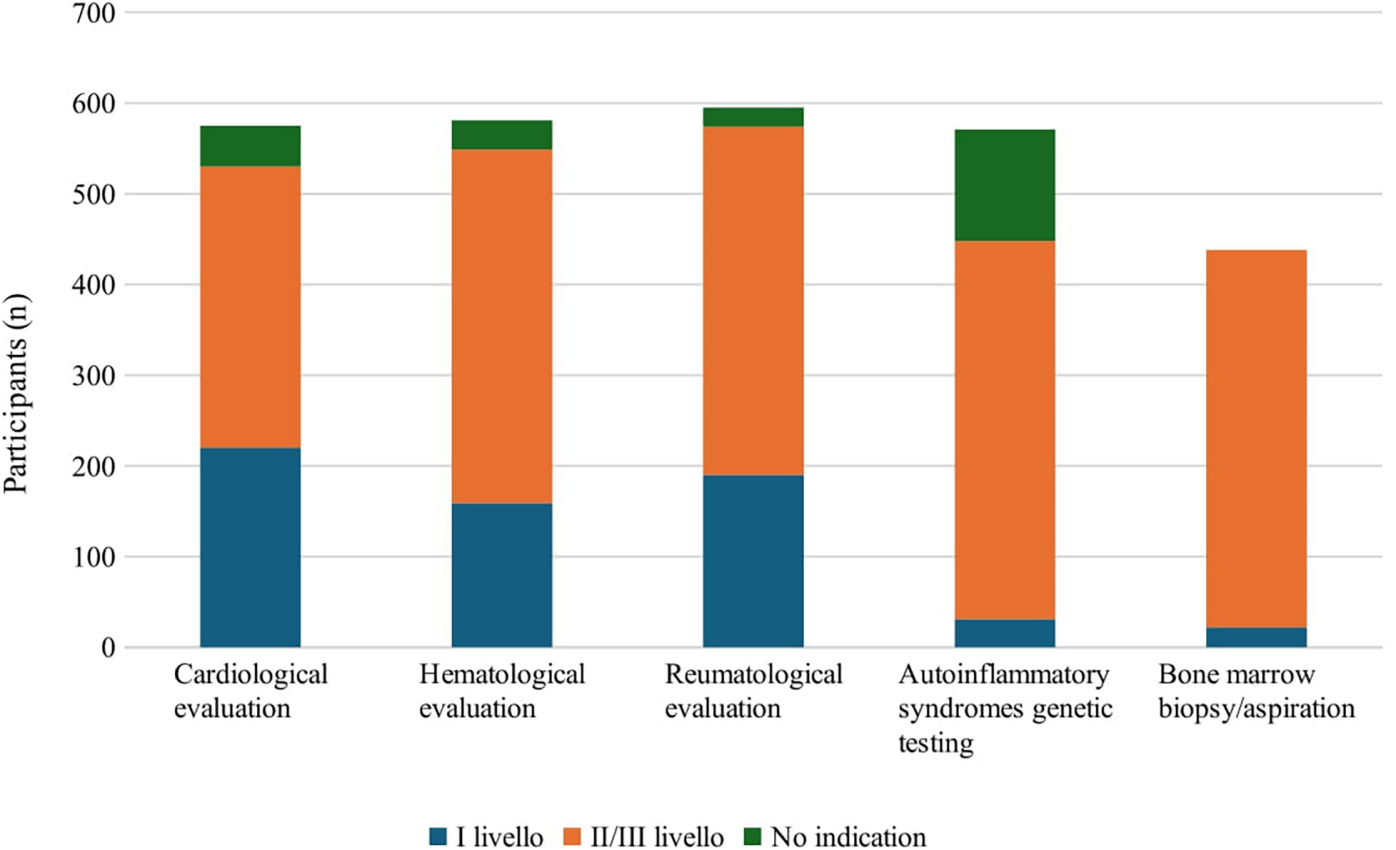
Specialist evaluations recommended by survey participants.

Eventually, participants would request specialist evaluations and consultations as second/third level investigations: 67.2% (417/620) would require genetic tests for autoinflammatory syndromes, 67% (416/620) bone marrow biopsy/aspiration, 62.9% (390/620) hematologic consultation, 61.9% (384/620) rheumatological consultation and 50% (310/620) cardiological evaluation (Figure 4).

### Treatment of FUO

**Figure 5:**
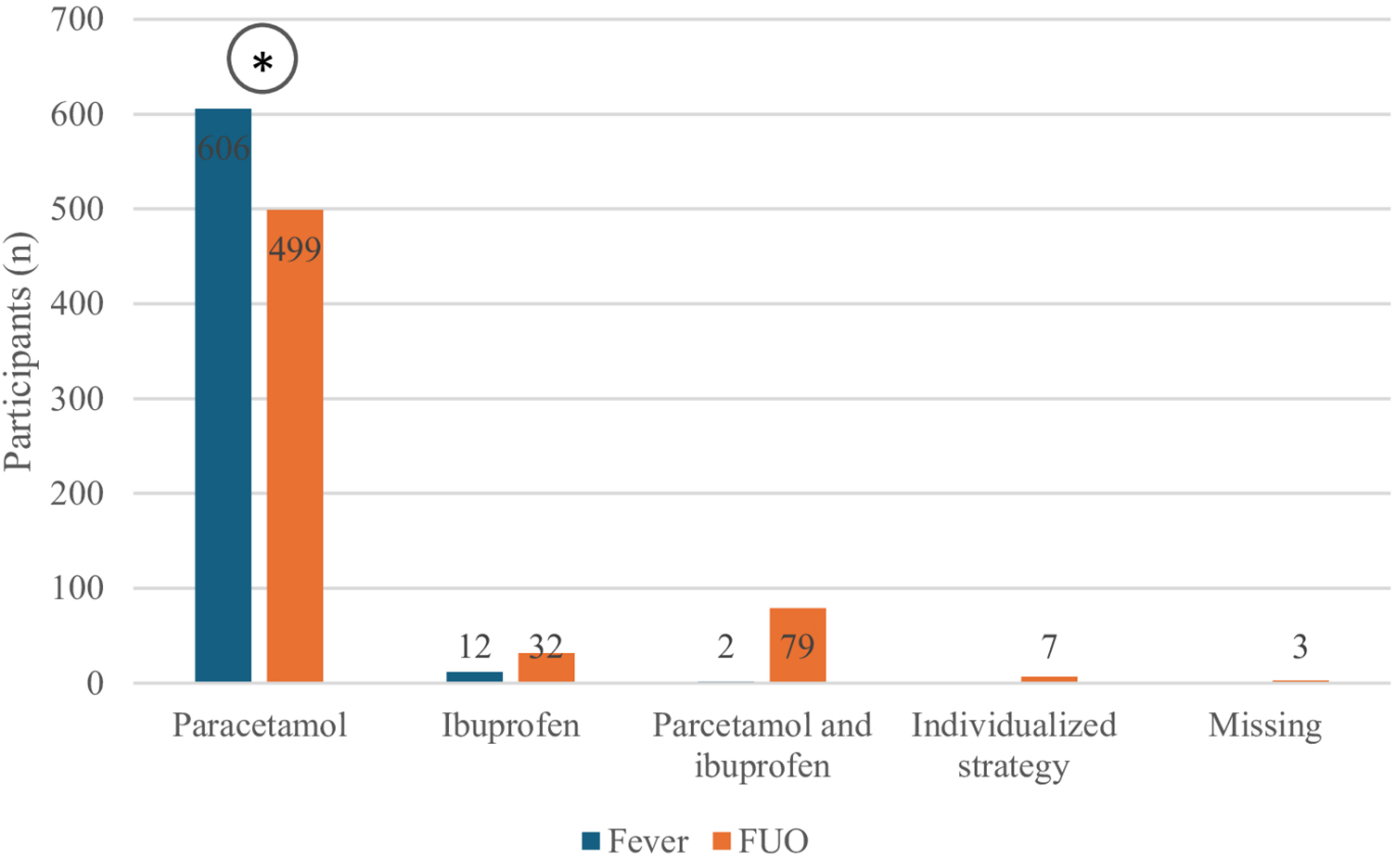
First-choice antipyretic in case of unspecified case of fever in children or FUO according to survey participants. *P < 0.0001.

In the treatment of FUO, 80.5% (499/620) of participants would prescribe paracetamol as the first-choice antipyretic (*P* < 0.0001 vs. others), 5.1% (32/620) would choose ibuprofen, and 12.7% (79/620) would use either paracetamol or ibuprofen without a specific preference. Compared to what the same participants reported for the treatment of children with non-prolonged fever, a significant decrease in the use of paracetamol as the first-choice drug was observed (80.5% vs. 97.7%; *P* < 0.0001), along with a corresponding but not significant increase in the use of ibuprofen (1.9% vs. 5.1%; *P* = 0.059). Additionally, we noticed a significant increase in the proportion of respondents declaring to use either paracetamol or ibuprofen without a specific preference (0.32% vs. 12.7%; *P* < 0.0001). Figure 5 shows the comparisons between the therapeutic approach to the febrile child in case of non-prolonged fever and FUO.

In case of persistent fever despite antipyretic administration in children with FUO, 44.2% (221/499) of those who prefer paracetamol would switch to ibuprofen, 27.6% (138/499) would continue prescribing paracetamol while checking and adjusting the dosage if needed, and 18.2% (91/499) would recommend alternating paracetamol and ibuprofen. Additionally, 4.0% (20/499) would switch to a combination of both paracetamol and ibuprofen, and 2% (10/499) would prescribe steroids. Only one participant suggested using physical methods.

Among those who prescribed ibuprofen as the first-choice drug, in case of persistent fever, 34.3% (11/32) would recommend alternating the two antipyretics, 31.2% (10/32) would continue with ibuprofen, eventually adjusting the dosage, 18.8% (6/32) would switch to paracetamol, 6.25% (2/32) would switch to a combination drug containing both paracetamol and ibuprofen, and 6.25% (2/32) would prescribe steroids.

**Figure 6:**
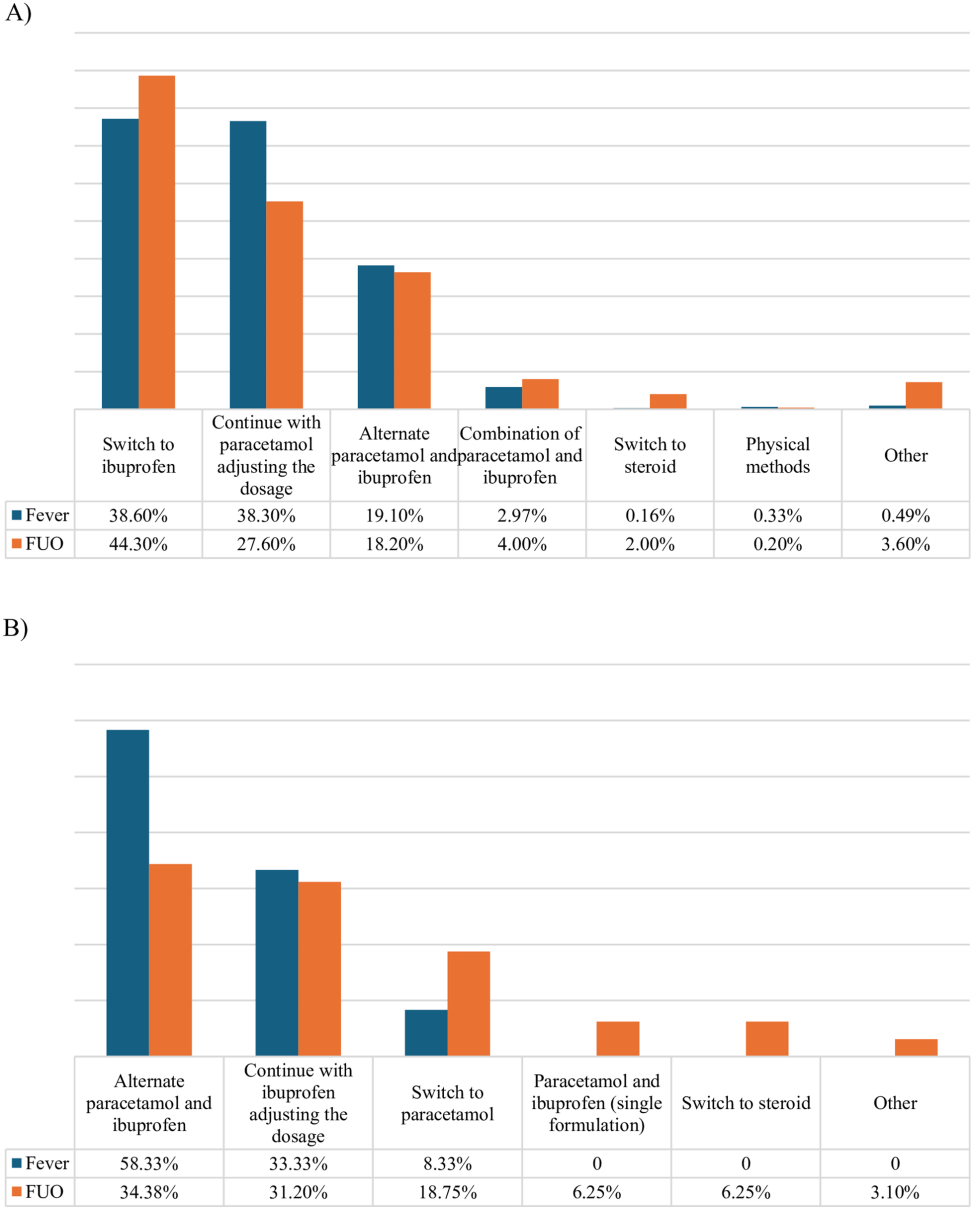
Therapeutic strategy to persistent fever. **(A)** Subgroup of participants who prescribe paracetamol as first-choice antipyretic. **(B)** Subgroup of participants who prescribe ibuprofen as first-choice antipyretic.

Figures 6 illustrate the comparison between approaches to persistent fever despite antipyretic administration in cases of regular fever and FUO, respectively, among those who recommend paracetamol (Figure 6A) as the first-choice drug and those who recommend ibuprofen (Figure 6B).

In case no etiology is identified once the diagnostic work-up is concluded, if fever persists 36.9% (229/620) of participants would continue only the symptomatic treatment of fever, 63% (391/620) declared to suggest an empiric treatment: 39% (242/620) would prescribe antibiotics, 13.7% (85/620) steroids and 4.5% (28/620) switch to a NSAID other than ibuprofen.

## Discussion

In the present study we surveyed 620 Italian pediatricians working in various clinical settings to analyze their approach to fever management and adherence to IFG (18) and other international guidelines (22–24). Furthermore, we investigated pediatricians’ knowledge and management of FUO, and proposed a shared pediatric definition.

With regard to fever treatment, our results confirmed those of one previous similar Italian survey (17), with about 97% of participants prescribing paracetamol as the first-choice antipyretic. Notably, none of the participants indicated other drugs (e.g.,steroids, metamizole) as first-choice drugs, according with guideline recommendations. In contrast with national and international guideline recommendations (18, 23, 24), 19.8% of interviewed pediatricians still suggested alternating paracetamol and ibuprofen for non-responsive fever.

On the other hand, the proportion of antipyretic prescription based on the presence of child's discomfort rather than on a sp ecific body temperature cut-off, as recommended by the guidelines, was almost doubled in comparison to 2018 survey (38.2% to 61.6%) (Table 3). We observed that correct behavior was more frequently reported by primary care practitioners compared to hospital pediatricians in this regard (69.5% vs. 56.0%; *P* = 0.0007). This difference may be attributed to the fact that antipyretics are often prescribed for hospitalized children above a certain body temperature threshold due to organizational protocols. The use of physical methods was largely abandoned.

**Table 3:** Management strategies of fever among pediatricians in the 2018 and 2024 surveys.

Strategy	2018 *n* (%)	2024 *n* (%)
First choice drug		
Paracetamol	546 (97.1)	606 (97.7)
Ibuprofen	12 (2.1)	12 (1.9)
Interchangeable	n.a.	2 (0.3)
Other drugs	4 (0.7)	0 (0)
Alternating use of antipyretics	69 (12.3)	123 (19.8)
Use of physical methods	289 (51.6)	2 (0.32)
Antipyretics according to discomfort	199 (38.2)	382 (61.6)
*Total*	*562*	*620*

Italic values denotes total number of survey participants.

Although most participants would not recommend administering antipyretics to prevent adverse events following vaccinations, still 16% of respondents suggest prophylactic use of antipyretics.

Various guidelines (25, 26) support prophylactic antipyretic use in case of anti-meningococcal B (MenB) vaccination, and a randomized controlled trial conducted on 4CMenB vaccine showed that prophylactic use of paracetamol reduced post-vaccination reactions without clinically relevant negative consequences on vaccine immunogenicity (27)*.* In our survey, however, only 1.6% of participants recommend the prophylactic use of antipyretics exclusively in children receiving MenB vaccination, whereas 41.9% would recommend it regardless of the type of vaccination, despite guidelines recommendations (18, 23, 24) and lack of robust evidence (28).

While most respondents correctly identified contraindications for ibuprofen and paracetamol, only 56% were aware of the age limitation for ibuprofen use in children over 3 months of age.

The most common reported contraindications for ibuprofen use were impaired renal function, and moderate or severe dehydration (13, 29). Varicella was also indicated as a contraindication by a consistent proportion of participants: accordingly, several studies reported an increased risk of complications (i.e., pneumonia, skin superinfection, necrotizing soft-tissue infection) in patients with varicella treated with NSAIDs (30, 31).

Another contraindication to the use of ibuprofen indicated by a small percentage of responders was Kawasaki disease (KD): the concomitant use of ibuprofen antagonizes the irreversible platelet inhibition induced by acetylsalicylic acid (32), therefore the 2017 American Heart Association guideline (33) advises against the concomitant use of ibuprofen in children with coronary aneurisms secondary to KD receiving acetylsalicylic acid.

Considering paracetamol, most participants were aware that its use is approved in children without any age limitation, including infants below 3 months old and neonates, and it can be administered in children at risk of dehydration (34). On the other hand, a small percentage considered paracetamol erroneously contraindicated in case of renal impaired function and gastrointestinal pathologies. The most frequent contraindication to the use of paracetamol according to responders was chronic hepatopathy. It is known that in case of overdose, paracetamol is metabolized to a toxic oxidative metabolite (N-acetyl-p-benzoquinoneimine). Paracetamol toxicity can result in cases of overdose or in some children with underlying conditions including chronic malnutrition or receiving specific concomitant medications (35) (36). A study investigating paracetamol metabolism and elimination in children with chronic liver dysfunction, suggested that there is no cause for concern in the use of single standard therapeutic dose in this patient group (37).

The latest section of the survey focused on pediatricians’ knowledge of FUO.

Moreover, a novel practical definition was elaborated by the expert panel and proposed to participants. Although 81% of agreement was reached, there was no unanimous consensus, and some observations were expressed. The main criticism concerned the proposed duration of continuous fever, since two weeks were considered too long a period for diagnosing FUO.

Considering this comment, a minimum duration of 7–10 days could be considered in an alternative definition. These controversies might be incorporated into future surveys or consensus documents.

Given the numerous potential etiologies, diagnosing FUO requires a comprehensive approach. The first step is the demonstration of fever by a healthcare professional, which also allows to rule out factitious fever. Regarding the subsequent diagnostic evaluation, there is currently no standardized algorithm available for the pediatric population, although various studies have elaborated possible diagnostic approaches.

A recent review (12) summarized literature findings on FUO, with a focus on possible diagnostic approaches. According to this data, in a child with a non-evocative history or physical examinations, first-level investigations should include preliminary laboratory tests (CBC, erythrocyte sedimentation rate, CRP, transaminases, LDH, IgG, IgA, IgM, blood and urine cultures) and early imaging (chest x-ray, abdominal ultrasound and echocardiogram with electrocardiogram). Subsequent second-level examinations should include specific viral and bacterial serologies and PCR tests; also, due to the increased incidence of tuberculosis, Quantiferon and/or Mantoux testing should be executed, especially in patients from endemic regions. When immune-mediated disorders are suspected, specific tests should be requested (e.g., ANA, ASO titer in at least two evaluations, anti-dsDNA, C3 and C4). Similarly, if IBD is suspected, ASCA and ANCA should be tested, along with fecal calprotectin. Lastly, some authors suggested that in selected cases genetic sequencing for autoinflammatory syndromes using Next Generation Sequencing (NGS) methods could be taken into account (38). Second-level instrumental investigations should be guided by the suspected district involved and included CT scan, MRI, scintigraphy, endoscopy, medullary aspirate, and tissue biopsies.

Our survey comprised most of the investigations already included in previous reviews (10, 12). Among the pediatricians participating in the survey, we observed a trend toward a greater agreement regarding first-level laboratory and instrumental tests, whereas more discrepancies arose on the examinations to be requested as second and third level investigations. However, it is important to underline that after the preliminary investigations, the choice of subsequent tests should be guided by diagnostic hypothesis: the investigations will vary depending on what emerges from the clinical history, physical examination, and initial assessments; therefore, it is not possible to identify a single panel of tests applicable to all situations. Our data combined with proposed diagnostic approaches from existing literature could be used in future studies to define a standardized diagnostic algorithm for FUO in pediatric patients, elaborating a step-by-step work-up differentiated upon the clinical suspicion, in order to avoid excessive and superfluous testing.

Considering antipyretic treatment, paracetamol was the first-choice drug in case of non-persistent fever for over 95% of participants, while in children with FUO this percentage, although still high, significantly decreased to 80%. However, in children with FUO the use of paracetamol might be preferred to avoid masking or delaying the diagnosis by using drugs with anti-inflammatory activity. Given the increasing attention to the rational use of medications (39, 40), it is interesting to note that 39% of participants would prescribe antibiotics, and 13.7% steroids, even with no certain diagnosis. Broad-spectrum antibiotics or steroids are the most commonly used medication in children with FUO. A Taiwan study (10) proposed a systematic approach based on a four-stage protocol, including therapeutic trials (empiric antimicrobial therapy, corticosteroids, intravenous immunoglobulins) in the last stage; 16.5% of 79 children received a therapeutic trial. However, empirical treatment may mask or delay the diagnosis of some infectious diseases or other severe conditions including cancer.

### Study limitations

The findings of this study must be considered in light of some limitations. Firstly, our dataset was limited, and the survey was administered indirectly by email. Given the low response rate, results may not fully reflect the general behavior of the entire population of Italian pediatricians. However, the final number of respondents, over 600, is equal or superior to the one reported in similar surveys published in literature (41–43). Secondly, the responses given in the questionnaire may not really reflect everyday clinical practice of the participants.

Due to length limitation of the questionnaire, answers were not stratified according to the child's age: it should be considered that the behavior of clinicians may vary between a febrile neonate or infant and an older child, due to the different risk of severe conditions. Finally, we cannot exclude some recall biases. For example, 4.2% of participants reported suspected bacterial superinfections in pediatric patients temporally related to ibuprofen use. However, this data relies solely on the participants’ memories, not on written medical records.

## Conclusions

Our study shows that pediatricians’ behaviors regarding the management of febrile child seem to have improved over time and are increasingly adhering to the national and international guidelines’ recommendations. However, some non-recommended behaviors, such as the alternating use of antipyretics, their use according to a given body temperature rather than the child's discomfort, and spread use to prevent adverse reactions following immunization, remain common. Our survey achieved an agreement of over 80% for a possible shared FUO definition as a continuous fever with daily peaks above 38°C, without apparent explanation for at least two consecutive weeks, documented on multiple circumstances by a healthcare professional.

Large agreement was observed on first-level laboratory and instrumental investigations in the diagnostic evaluation of FUO, whereas more discrepancies arose on second and third-level investigations. Discrepancies were observed also in the use of therapeutic trials with antibiotics, steroids or NSAIDs.

Compared to what participants reported for the treatment of non-prolonged fever, a significant decrease in the prescription of paracetamol as first-choice drug in children with FUO was observed.

The management of children with FUO remains problematic. suggesting the need of educational interventions, and evidence-based consensus documents.

## Data Availability

The raw data supporting the conclusions of this article will be made available by the authors, without undue reservation.

## Supplementary material

Supplementary Table S1 Contraindications to the use of antipyretics according to survey participants (n = 529).

Supplementary Table S2 Suspected infectious complications temporally related to the use of ibuprofen, as reported by participants (n=26/620).

Supplementary Figure S1 Indications to antipyretic prescription according to survey participants. (T = body temperature).

Supplementary Figure S2 Vaccines for which prophylactic antipyretic administration is recommended by pediatricians participating in the survey (n=105): DTP = Diphtheria, Tetanus, acellular Pertussis vaccine, PCVs = Pneumococcal Conjugate Vaccines, MenB = Meningococcal B vaccine, MMR/MMRV = Measles, Mumps, and Rubella/ Measles, Mumps, Rubella, and Varicella.

Supplementary Figure S3 Level of agreement of survey participants to the proposed definition of FUO (Likert Scale).

Supplementary Figure S4 Therapeutic approach to persistent fever in case of unidentified cause following the diagnostic work-up.
