# Supplementary material for: Fever management in children and insights into fever of unknown origin: a survey among Italian pediatricians
Source: Front Pediatr. 2024 Nov 1;12:1452226. doi: 10.3389/fped.2024.1452226 (PMC11563795; doi:10.3389/fped.2024.1452226)
Supplement: Supplementary Table S1 [file Table1.pdf]

Table S1. Contraindications to the use of antipyretics according to survey participants (n=529)

	Paracetamol (n, %)	Ibuprofen (n, %)	P
Suspected/confirmed impaired renal function	15 (2.4)	398 (62.7)	<0.0001
Moderate/severe dehydration	10 (1.6)	362 (58.3)	<0.0001
Chronic hepatopathy	254 (40.9)	104 (16.7)	<0.0001
Infants < 3 months	2 (0.3)	353 (56.9)	<0.0001
Hemorrhagic diathesis	9 (1.4)	286 (46.1)	<0.0001
Gastrointestinal pathologies (i.e. diarrhea)	2 (0.3)	236 (38.0)	<0.0001
Varicella	3 (0.4)	277 (44.6)	<0.0001
Other herpetic infections	1 (0.16)	102 (16.4)	<0.0001
Kawasaki disease during treatment	7 (1.1)	101 (16.2)	<0.0001
Allergic asthma (excluding drug induced forms)	3 (0.4)	40 (6.4)	<0.0001
Acute otitis media	4 (0.6)	12 (1.9)	0.0749
Pneumonia	1 (0.1)	46 (7.4)	<0.0001
