# Supplementary material for: Fever management in children and insights into fever of unknown origin: a survey among Italian pediatricians
Source: Front Pediatr. 2024 Nov 1;12:1452226. doi: 10.3389/fped.2024.1452226 (PMC11563795; doi:10.3389/fped.2024.1452226)
Supplement: Supplementary Table S2 [file Table2.pdf]

Table S2. Suspected infectious complications temporally related to the use of ibuprofen, as reported by participants (n=26/620)

	Participants (n, %)
Infectious complications	26 (4.2%)
Suspected of confirmed bacterial pneumonia (not complicated)	6
Complicated pneumonia (pleural empyema, pulmonary abscess or necrotizing pneumonia)	9
Otomastoiditis	4
Fascitis/necrotizing fasciitis	2
Bacterial superinfection of varicella lesions	3
Cellulitis	2
