# Supplementary figures and images for: Fever management in children and insights into fever of unknown origin: a survey among Italian pediatricians
Source: Front Pediatr. 2024 Nov 1;12:1452226. doi: 10.3389/fped.2024.1452226 (PMC11563795; doi:10.3389/fped.2024.1452226)

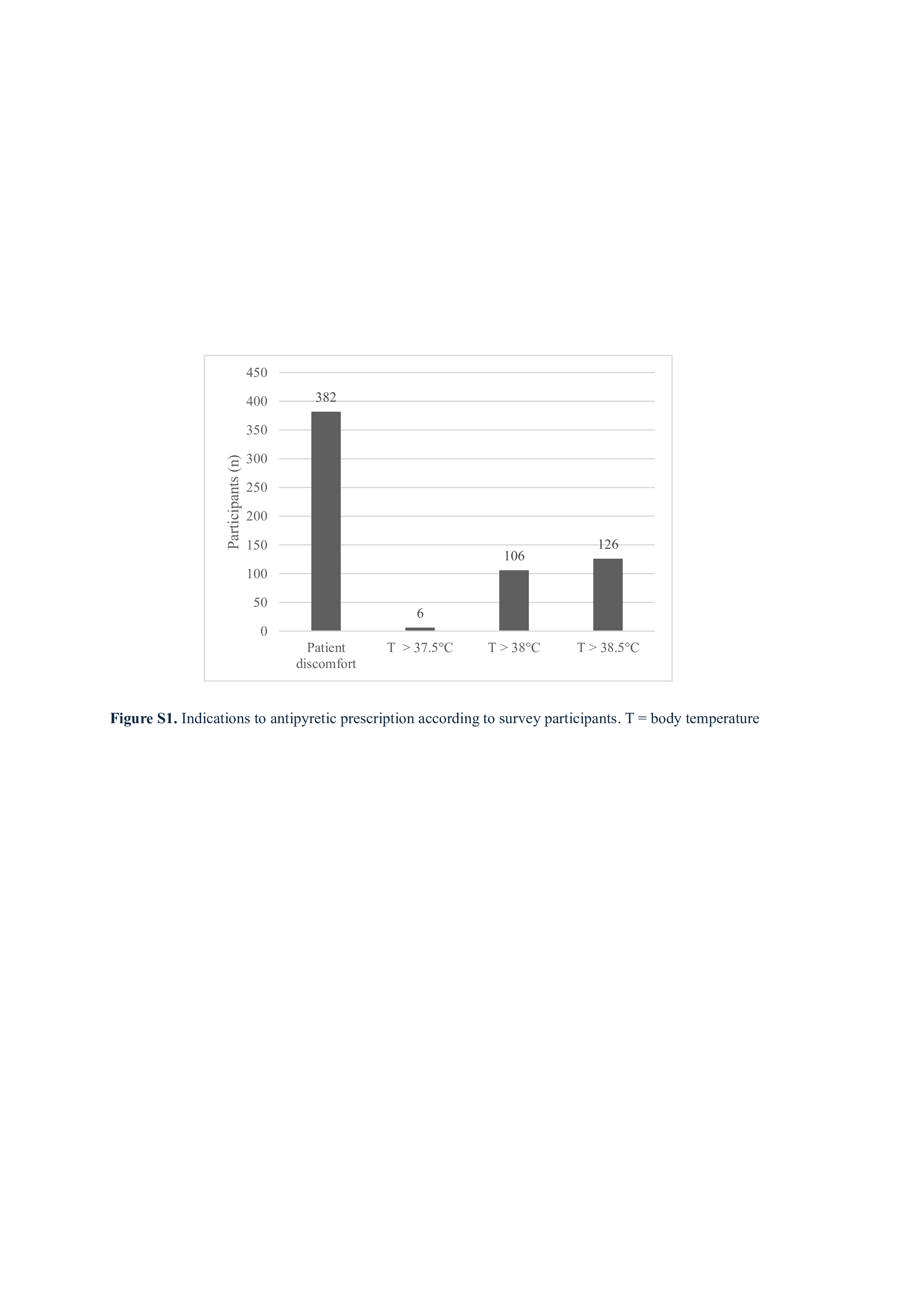

Supplement: Supplementary Figure S1 [file Image1.jpeg]

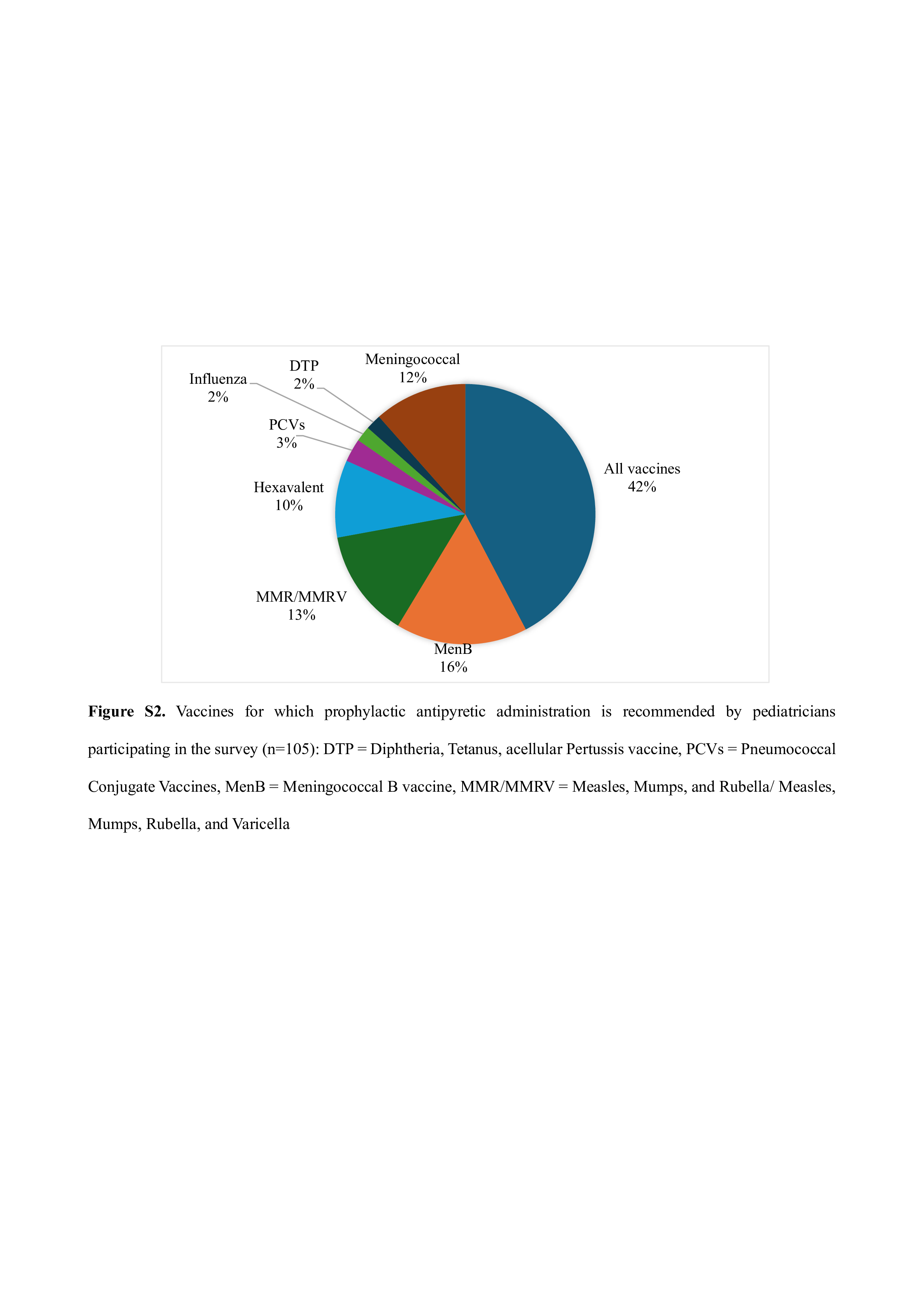

Supplement: Supplementary Figure S2 [file Image2.jpeg]

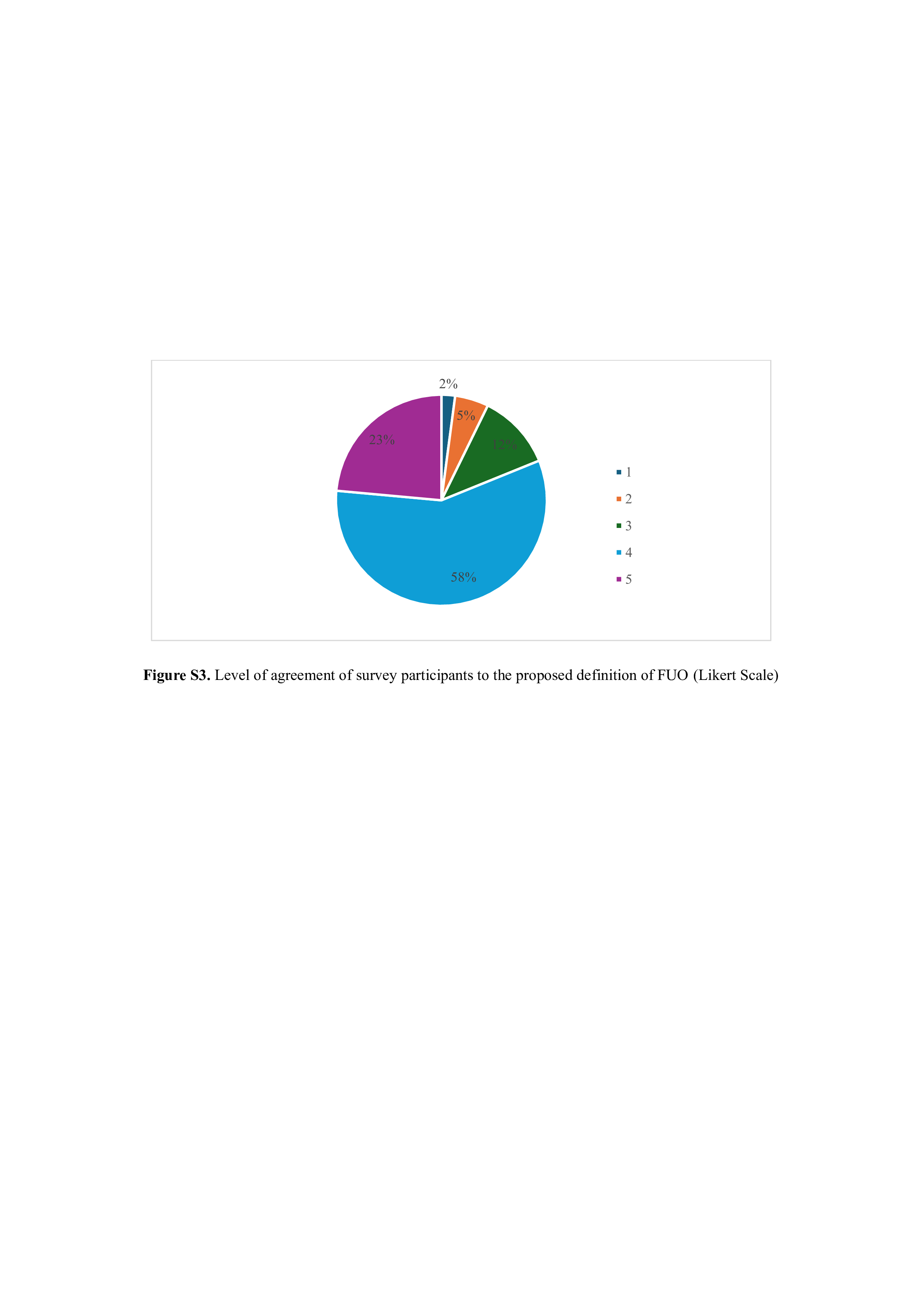

Supplement: Supplementary Figure S3 [file Image3.jpeg]

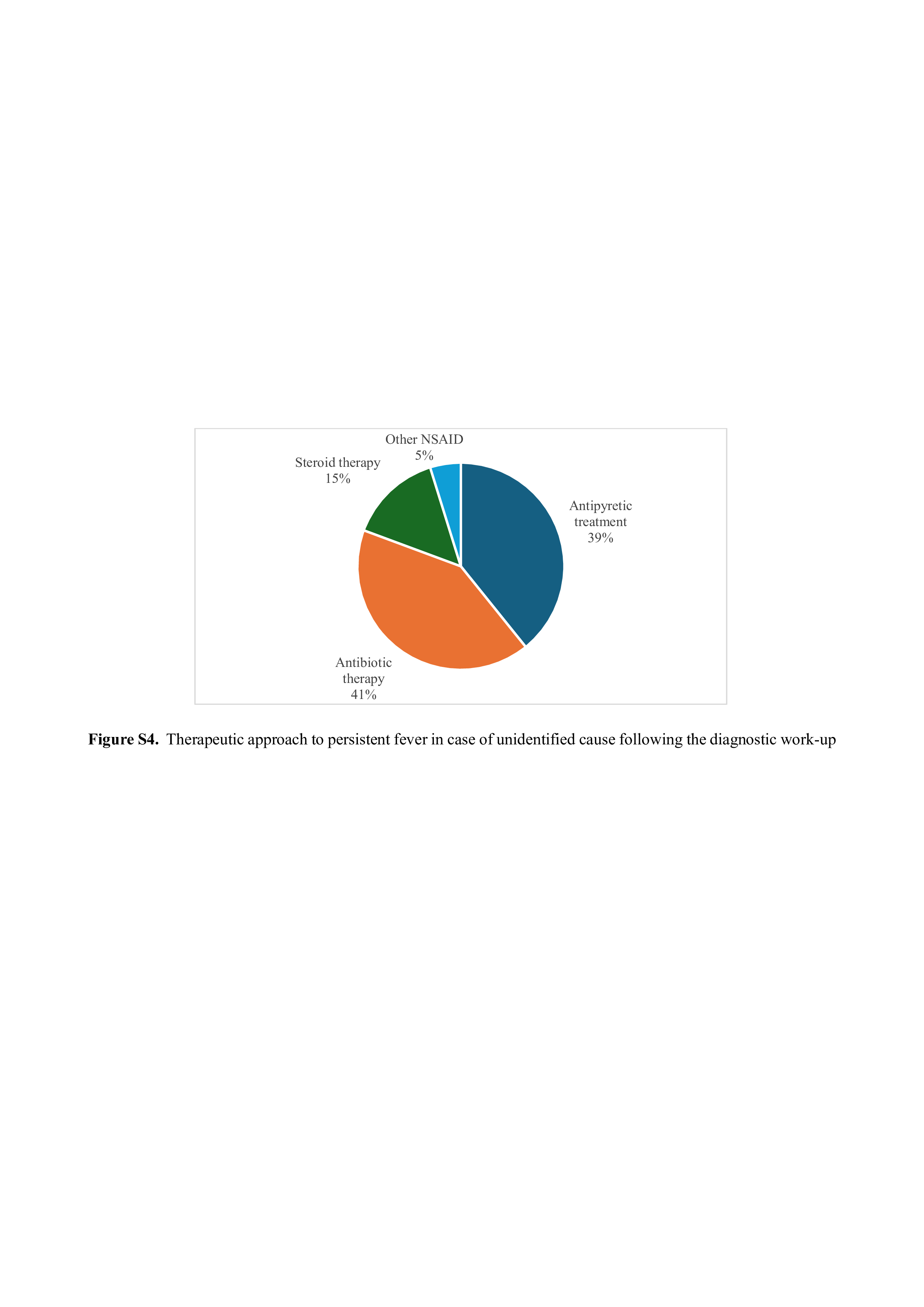

Supplement: Supplementary Figure S4 [file Image4.jpeg]
