# Supplementary material for: Fever management in children and insights into fever of unknown origin: a survey among Italian pediatricians
Source: Front Pediatr. 2024 Nov 1;12:1452226. doi: 10.3389/fped.2024.1452226 (PMC11563795; doi:10.3389/fped.2024.1452226)
Supplement: Supplementary file 7 [file Datasheet1.pdf]

Supplementary Material A - Survey questionnaire

1. Region of provenience

2. Province of provenience

3. Work setting
    a. Primary care pediatrician
    b. University Hospital
    c. Pediatric University Hospital
    d. Non-University Hospital
    e. Pediatric non-University Hospital
    f. Altro

4. Age

5. Years of work experience as Pediatrician
    a. 0-10 years
    b. 11-20 years
    c. 21-30 years
    d. Over 30 years

6. In your clinical practice, which antipyretic do you usually use as first choice in children?
    a. Paracetamol
    b. Ibuprofen
    c. Either one or the other

7. What do you recommend in case of fever not responding to the first-choice antipyretic (paracetamol)?
    a. Physical methods
    b. Continue with paracetamol, checking the prescribed dosage
    c. Switch to ibupofen
    d. Recommend alternating paracetamol and ibuprofen
    e. Switch to a combination of paracetamol and ibuprofene in two different formulations
    f. Switch to a combination of paracetamol and ibuprofene in a single formulation
    g. Switch to steroids
    h. Other

8. What do you recommend in case of fever not responding to the first-choice antipyretic (ibuprofen)?
    a. Continue with ibuprofen, checking the prescribed dosage
    b. Switch to paracetamol
    c. Recommend alternating paracetamol and ibuprofen

9. Do you use an antipyretic for preventing fever and discomfort related to vaccinations?
    a. No
    b. Yes, ibuprofen
    c. Yes, paracetamol
    d. Other
10. For what type of vaccinations?
    a. Influenza

b. Diphtheria, Tetanus, acelluar Pertussis (DTP)
c. Hexavalent
d. Meningococcal
e. Meningococcal B
f. Measles, Mumps and Rubella/Measles, Mumps, Rubella and Varicella (MMR/MMRV)
g. All vaccinations

11. Which antipyretic do you prescribe to treat fever or discomfort following a vaccination?
    a. Paracetamol
    b. Ibuprofen
    c. None
    d. Other

12. When do you prescribe antipyretics in a child with fever?
    a. Regardless of the presence of discomfort, in case of febrile body temperature
    b. According to the presence of discomfort, regardless of body temperature

13. In case of answer a. to the previous question, above what body temperature values?
    a. > 37.5°C
    b. > 38°C
    c. > 38.5°C

14. Are you aware of any clinical situations in which ibuprofen prescription is not recommended?
    a. Yes
    b. No

15. In case of answer Yes to the previous question, in what clinical situation?
    a. Suspected/confirmed impaired renal function
    b. Moderate/severe dehydration
    c. Infants < 3 months
    d. Hemorrhagic diathesis
    e. Varicella infection
    f. Gastrointestinal pathologies (i.e. diarrhea)
    g. Chronic epathopaty
    h. Other herpetic infections
    i. Kawasaki disease during treatment
    j. Pneumonia
    k. Allergic asthma (except allergy to NSAIDs)
    l. Upper respiratory tract infections (URTI)
    m. Lower respiratory tract infections (LRTI)
    n. Skin infections
    o. Acute otitis media
    p. Other

16. Are you aware of any clinical situations in which paracetamol prescription is not recommended?
    a. Yes
    b. No

17. In case of answer Yes to the previous question, in what clinical situation?
    a. Chronic epathopaty
    b. Suspected/confirmed impaired renal function
    c. Moderate/severe dehydration
    d. Infants < 3 months
    e. Hemorrhagic diathesis
    f. Varicella infection
    g. Gastrointestinal pathologies (i.e. diarrhea)
    h. Other herpetic infections
    i. Kawasaki disease during treatment
    j. Pneumonia
    k. Allergic asthma (except allergy to paracetamol)
    l. Lower respiratory tract infections (LRTI)
    m. Skin infections
    n. Acute otitis media
    o. Other

18. In your clinical practice over the past few years, have you suspected cases of complications of infectious diseases associated with the use of NSAIDs?
    a. Yes
    b. No

19. In what cases?

20. In your clinical practice, which of the folllwing definition of FUO do you apply?
    A. Fever (> 38°C) persisting for at least 3 weeks, in a patient who underwent routine medical investigations exclusively in a hospital setting, without the cause being determined
    B. Two or more weeks of fever (> 38.5°C), measured on at least 4 different occasions
    C. Febrile illness for which the cause could not be clarified during at least 3 weeks of outpatient evaluation, or for more than one week of hospital observation
    D. Persistence of fever for more than 8 days in a child whose medical history, examination, and laboratory tests do not reveal a possible cause

21. How many FUO cases have you diagnosed over the last year?
    a. 0 or 1
    b. 2 - 5
    c. 6 - 10
    d. Over 10

22-23. Which diagnostic criteria do you consider for diagnosising FUO?

22. Body temperature measured by a healthcare professional in multiple occasions
    a. > 37.5°C
    b. > 38°C
    c. > 38.5°C

23. Fever duration for a continouous period of:
    a. > 1 week
    b. > 2 weeks

c. > 3 weeks

24. Setting of body temperature measurement and repeated clinical evaluation:
    a. Outpatient
    b. Hospital
    c. Outpatient or hospital
    d. Other

25. Per una corretta diagnosi ritieni che sia necessario in ogni caso il ricovero ospedaliero al fine di documentare la presenza e il grado di febbre?
    a. Si
    b. No

26. Rate from 1 to 5 your agreement to the following new definition of FUO in the pediatric age (1 = strongly disagree, 5 = fully agree):

    "Continuous fever, with daily peaks above 38°C, without apparent explanation, documented by a healthcare professional * for at least two consecutive weeks.

    **Fever should be documented by a healthcare professional on multiple occasions in hospital setting, or at least three times in different days in outpatient setting."*

27. In case of rating between 1 and 3, explain your criticisms.

28. Beside a thorough medical history and accurate physical examination, which of the following laboratory test would you recommend in a child with FUO as first or second/third level examinations?
    a. Complete blood count
    b. Liver enzyme
    c. Renal function
    d. Coagulation
    e. Lactate dehydrogenase (LDH)
    f. Uricemia
    g. Ferritine
    h. Thyroid hormones
    i. Urine exam
    j. Pro-BNP
    k. Creatin phosphokinase
    l. Anti-nuclear antibodies (ANA)
    m. Extractable Nuclear Antigen Antibodies (ENA)
    n. C3, C4
    o. Protidogram
    p. Lymphocyte subpopulation
    q. IgG, IgA, IgM
    r. Peripheric blood smear
    s. Fecal calprotectine
    t. Lipid panel

29. Which of the following microbiological tests would you recommend?
    a. Urine culture
    b. Stool culture
    c. Blood culture
    d. Mantoux or Quantiferon test
    e. Cytomegalovirus PCR or serology
    f. Epstein-Barr Virus PCR or serology
    g. Bartonella serology
    h. Tularemia serology
    i. Listeria serology
    j. ì Leishmania PCR or serology
    k. Leptospira PCR, urinary antigen or serology
    l. Legionella urinary antigen
    m. Respiratory virus, Mycoplasma and Chlamydia PCR on nasopharyngeal swab
    n. Rapid antigen detection test (RAD) for group A beta-hemolytic Streptococcus pyogenes
    o. HIV testing
    p. Parasitological stool research
    q. Covid-19 PCR swab

30. Which of the following instrumental tests would you recommend?
    a. Abdominal ultrasound
    b. Chest X-Ray
    c. Paranasal sinuses X-Ray
    d. Chest CT
    e. Abdomen/pelvis CT
    f. Brain/paranasal sinuses CT
    g. Bowel ultrasound
    h. Brain MRI
    i. Whole-body MRI
    j. Lung ultrasound

31. Which of the following specialist evaluations would you recommend?
    a. Cardiological evaluation
    b. Hematological evaluation
    c. Rheumatological evaluation
    d. Autoinflammatory syndromes genetic testing
    e. Bone marrow biopsy/aspiration

32. What further investigations would you request?

33. What is your antipyretic of choice for fever treatment in a child with FUO?
    a. Exclusively paracetamol
    b. Exclusively ibuprofen
    c. Paracetamol and ibuprofen
    d. Other

34. What do you recommend in case of persistent fever despite antipyretic (paracetamol) administration in a child with FUO?
    a. Continue with paracetamol, checking the prescribed dosage
    b. Switch to ibupofen
    c. Alternate use of paracetamol and ibuprofen
    d. Switch to a combination of paracetamol and ibuprofene in two different formulations
    e. Switch to a combination of paracetamol and ibuprofene in a single formulation
    f. Switch to steroids
    g. Other

35. What do you recommend in case of persistent fever despite antipyretic (ibuprofen) administration in a child with FUO?
    a. Continue with ibuprofen, checking the prescribed dosage
    b. Switch to paracetamol
    c. Switch to a combination of paracetamol and ibuprofene in a single formulation
    d. Other

36. In case no etiology is identified once the diagnostic work-up is concluded and fever still persisted, would you recommend any pharmacological treatment?
    a. Yes, with antibiotics
    b. No, continue only with symptomatic treatment of fever
    c. Yes, with steroids
    d. Yes, with other FANS different from ibuprofen
    e. Other

37. In light of recent Italian consensus papers highlighting the role of paracetamol as first-choice antipyretic and underlining the reduction of child's discomfort as primary goal of antipyretic therapy, would you routinely recommend paracetamol as first-line treatment in febrile children, considering also the high safety profile and efficacy, especially in case of dehydration and bacterial superinfection risk?
    a. Yes
    b. No
